# Medical students as EMTs: skill building, confidence and professional formation

**DOI:** 10.3402/meo.v19.24829

**Published:** 2014-07-22

**Authors:** Thomas Kwiatkowski, William Rennie, Alice Fornari, Salaahuddin Akbar

**Affiliations:** 1 Department of Emergency Medicine, Hofstra North Shore-LIJ School of Medicine, Hofstra University, Hempstead, NY, USA; 2 Department of Science Education, Population Health, and Family Medicine, Hofstra North Shore-LIJ School of Medicine, Hofstra University, Hempstead, NY, USA; 3 Center for Learning and Innovation, North Shore-LIJ Health System, New Hyde Park, NY, USA

**Keywords:** undergraduate medical education, curriculum, pre-hospital care, clinical skills, team building, professional role

## Abstract

**Objective:**

The first course of the medical curriculum at the Hofstra North Shore-LIJ School of Medicine, *From the Person to the Professional: Challenges, Privileges and Responsibilities*, provides an innovative early clinical immersion. The course content specific to the Emergency Medical Technician (EMT) curriculum was developed using the New York State Emergency Medical Technician curriculum. Students gain early legitimate clinical experience and practice clinical skills as team members in the pre-hospital environment. We hypothesized this novel curriculum would increase students’ confidence in their ability to perform patient care skills and enhance students’ comfort with team-building skills early in their training.

**Methods:**

Quantitative and qualitative data were collected from first-year medical students (*n*=97) through a survey developed to assess students’ confidence in patient care and team-building skills. The survey was completed prior to medical school, during the final week of the course, and at the end of their first year. A paired-samples *t*-test was conducted to compare self-ratings on 12 patient care and 12 team-building skills before and after the course, and a theme analysis was conducted to examine open-ended responses.

**Results:**

Following the course, student confidence in patient care skills showed a significant increase from baseline (*p*<0.05) for all identified skills. Student confidence in team-building skills showed a significant increase (*p*<0.05) in 4 of the 12 identified skills. By the end of the first year, 84% of the first-year students reported the EMT curriculum had ‘some impact’ to ‘great impact’ on their patient care skills, while 72% reported the EMT curriculum had ‘some impact’ to ‘great impact’ on their team-building skills.

**Conclusions:**

The incorporation of EMT training early in a medical school curriculum provides students with meaningful clinical experiences that increase their self-reported level of confidence in the performance of patient care skills early in their medical education.

In their landmark publication, Cooke et al. (1) outline four goals of medical education. One of these goals specifically addresses clinical education, stating that ‘students should experience integration of skills and knowledge in a way that prepares them for practice’. The authors further recommend that medical students be given some form of early clinical immersion. The first course of the medical curriculum at the Hofstra North Shore-LIJ School of Medicine (SOM), *From the Person to the Professional: Challenges, Privileges and Responsibilities* (CPR), addresses the goal of integrating knowledge and skills and provides an innovative early clinical immersion to foster this connection. The course content specific to the Emergency Medical Technician (EMT) curricular component was developed using the New York State Department of Health Emergency Medical Technician curriculum with added elements of the basic biomedical, social, and clinical science content essential for medical student education. Students gain clinical experience through the supervised practice of skills as functional team members in the pre-hospital environment.

This approach to ‘knowledge in action’ promotes the early transformation of students from medical bystanders to active participants in meaningful patient encounters. Within the first few weeks of school, students learn to assess patients with a wide variety of medical, traumatic, and behavioral problems, while also learning appropriate cognitive, communication, interventional, and team-based skills. They learn how to interact effectively with patients of diverse ages and social backgrounds and with a variety of health care professionals, both in the field and in the hospital.

A review of the literature found only a few papers that describe integration of an EMT curriculum into undergraduate medical education, with virtually nothing reported on this topic in the past 25 years. Harrison et al. (2) developed an EMT course for first-year medical students, which led to eligibility for EMT certification. The author concluded that for the course to be successful, it would have to have equal status to other courses and be scheduled during normal curricular hours. Bradley et al. (3) focused on redesigning an EMT curriculum to meet the needs of medical students. It was concluded that reducing the standard EMT curricular content hours would encourage medical schools to incorporate EMT certification into their curriculum. Burdick and Davidson (4) reported on a required EMT course for medical students to help meet the goals of their ‘Introduction to Clinical Medicine’ course. Brown and Zimitat (5) reported on providing second-year medical students the opportunity to be part of ambulance ‘ridealongs’, exposing students to clinical activities *in situ*. Our research found no publications directly related to the Hofstra North Shore-LIJ School of Medicine curricular model, which incorporates an EMT curriculum leading to NYS certification into the first course students experience as they begin their medical education.

The purpose of this manuscript is to report on the impact of our early meaningful clinical experience that provides a legitimate role for our students and builds our students’ level of confidence in patient care and teambuilding skills longitudinally over the first year of their education. We hypothesized that beyond the development of an early clinical approach to patients, this novel course would 1) increase students’ confidence in their ability to perform patient care skills in multiple care settings and 2) that early exposure to patient care, through involvement with Emergency Medical Services (EMS), would enhance students’ comfort with team-building skills early in their training.

## Methods

### Study design

This is a causal study design that includes participation from all of participants in the population. This type of research is used to measure what impact the EMT curriculum had on existing norms and assumptions. Key outcomes measures include patient care skills and team-building skills. This survey had a reflective component as it was looking back on the students’ perceptions of their skill development over the first year of medical school.

### Population & setting

The Hofstra North Shore-LIJ School of Medicine curriculum combines basic science with hands-on clinical experiences. It stresses an integrated, team-based model of learning and patient care with frequent opportunities for skills practice and self-reflection on students’ development.

The EMT experience begins on the first day of medical school and gives students their first immersion into clinical practice. The curriculum is delivered in large group didactics, small group workshops, through high fidelity simulation and most importantly in the community, through active participation in the care of acutely ill patients as part of a team of health care professionals in the pre-hospital environment. At the conclusion of this course, students sit for the New York State EMT certification examination, which allows them to function independently as EMTs. The North Shore-LIJ Health System's Center for Emergency Medical Services provides the opportunity for student involvement in real-life clinical care.

During the 9-week course, students are required to complete on average four ambulance tours on Health System ambulances. These tours allow students to enter local communities and encounter patients in their homes where they actively participate in evaluating, treating and transporting the patients together with certified EMTs and paramedics. Students also participate in 911 and inter-facility transports. Subsequent to this initial course, students continue serving as NYS Certified EMTs on additional ambulance tours during the remainder of their first year.

All 100 students enrolled in the SOM year 1 and year 2 classes were invited to participate in this study. Three students were excluded because they did not submit the Time 1 survey, accounting for the sample size of 97 students.

### Survey development

Development of a new survey instrument was required as there was no previous survey in the literature. Survey development and validation was an integrated process. Our first step included a literature search to determine what patient care skills and team collaboration skills are traditionally expected of first- and second-year medical students.

Our next steps focused more on validation of the content included in the survey. Representatives from the SOM and the North Shore-LIJ Health System clinical emergency medicine faculty reviewed the patient care and team-building skills, confirmed they were appropriate, and shared further suggestions for the survey.

Next, we identified a convenience sample of Health System residents, who were certified EMTs prior to entering medical school, as ‘key informants’ to complete an electronic questionnaire to validate the patient care and team-building skills we listed on our survey. These responses informed the skills that we ultimately included in our survey.

Fourth, we reviewed the learning objectives from the CPR course to identify patient care and team-building skills that are included in the CPR course curriculum to ensure they were aligned with our survey items.

This survey (see Supplementary file, survey 1) was used for both the Time 1 survey (prior to medical school) and Time 2 (9 weeks at the end of the CPR course).

The Time 3 survey (see Supplementary file, survey 2) (distributed at 50 weeks) was developed by the research team to identify the impact of the students’ longitudinal EMT experience, specifically addressing patient care and team-building skills over their first year of medical school.

### Survey distribution

This study used a survey methodology that collected pre/post quantitative data as well as open-ended responses to capture students’ perceptions of their confidence in patient care and team-building skills. This study was approved by the Hofstra North Shore-LIJ School of Medicine Institutional Review Board.

All surveys were distributed electronically. The Time 1 (prior to medical school) and Time 2 (9 weeks) survey included self-ratings in confidence levels of 12 patient care skills and 12 team-building skills as well as global self-assessment questions of students’ confidence in both patient care skills and team-building skills (Tables 1 and 2). We distributed this survey to students immediately prior to coming to medical school (Time 1) and 9 weeks into medical school, upon receiving their NYS EMT certification (Time 2).

**Table 1:** Self-reported confidence in patient care skills (*N*=97)

Survey questions	Pre	Post^a^	Mean differential	*P*
Approaching patients	2.95	3.53	0.58	<0.05
Listening to patients	3.34	3.73	0.39	<0.05
Using appropriate body language	3.02	3.63	0.61	<0.05
Establishing rapport with patients	3.02	3.52	0.49	<0.05
Obtaining a medical history	2.02	3.13	1.11	<0.05
Using appropriate interview styles with patients	2.13	3.18	1.04	<0.05
Understanding a patient's perspective	2.94	3.47	0.54	<0.05
Conducting a basic physical exam	1.55	2.41	.87	<0.05
Responding to a patient's medical issues	1.74	2.71	.97	<0.05
Responding to a patient's psychosocial issues	1.84	2.60	0.76	<0.05
Managing time in individual patient encounters	2.09	2.65	0.56	<0.05
Identifying clinical dilemmas that require ethical decision making	2.31	2.87	0.56	<0.05
On a scale of 1–5, how much confidence do you have right now in patient care skills?	2.74	3.54	0.79	<0.05

a Pre is prior to beginning medical school and post is at end of the CPR course (9 weeks).

We distributed the Time 3 survey (at 50 weeks into medical school) to identify the impact of the students’ longitudinal EMT experience.

## Results

The age of students enrolled in the first two classes ranged from 21 to 46 years. Forty-six males and 51 females participated in this study. Thirteen (13%) of the students were certified EMTs prior to entering medical school.

### Quantitative data

All data are reported as pre/post comparisons of the students’ perceived levels of confidence in patient care and team-building skills at Time 1(baseline) and Time 2 (9 weeks). Following the course, student confidence in patient care skills showed a significant increase from Time 1 results (*p*<0.05) for 11 of the 12 identified skills (Table 1). Student confidence in team-building skills showed a significant increase (*p*<0.05) in four of the 12 identified skills (Table 2). There was a significant difference in the pre (*M*=2.74, SD=0.72) and post (*M*=3.54, SD=0.79) scores for global patient care skills; *t*(96)=7.74, *P*<0.05 (Table 1). There was no significant difference in the pre- (*M*=3.98, SD=0.79) and post- (*M*=4.12, SD=0.69) scores for global team-building skills. However, when we excluded the students who reported prior EMT training (*n*=13) the results were significant for increased confidence in global team-building skills (Table 2).

**Table 2:** Self-reported confidence in team building skills (*N*=97)

Survey questions	Pre	Post^a^	Mean differential	*P*
Understanding my role and responsibilities as a medical student	2.65	3.14	0.49	<0.05
Understanding the roles and responsibilities of others	2.59	3.18	0.59	<0.05
Developing a respectful working alliance	3.56	3.62	0.06	0.43
Setting common goals	3.42	3.49	0.07	0.31
Understanding the strengths of different team members	3.24	3.44	0.21	<0.05
Decision making	2.99	3.14	0.15	0.06
Adapting to a situation	3.47	3.35	−0.12	0.08
Being flexible	3.57	3.49	−0.07	0.29
Anticipating the needs of other team members	3.00	3.07	0.07	0.38
Prioritizing work	3.30	3.16	0.13	0.11
Conflict management	3.08	3.10	0.02	0.78
Trusting other team members	2.98	3.22	0.24	<0.05
On a scale of 1–5, how much confidence do you have right now in team-building skills?	3.98	4.12	0.14	.07

a Pre is prior to beginning medical school and post is at end of CPR course (9 weeks).

**Fig. 1:**
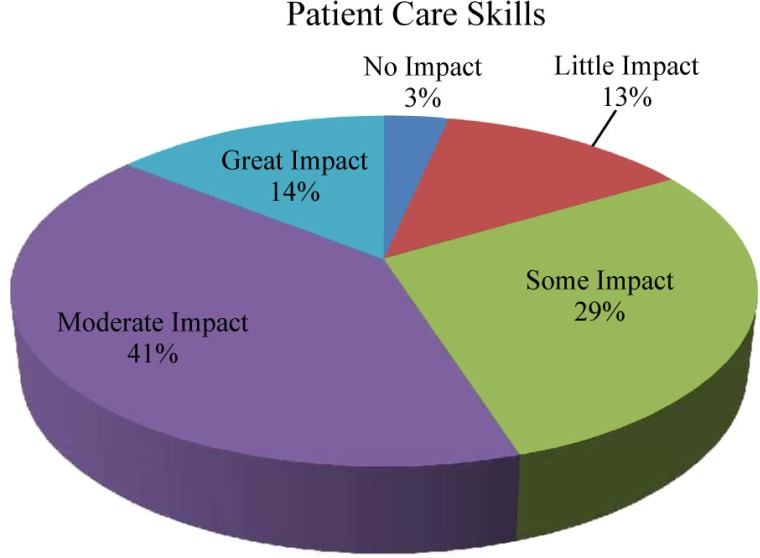
Self-reported impact of the EMT curriculum and ambulance experiences on students’ patient care skills (*N*=97).

**Fig. 2:**
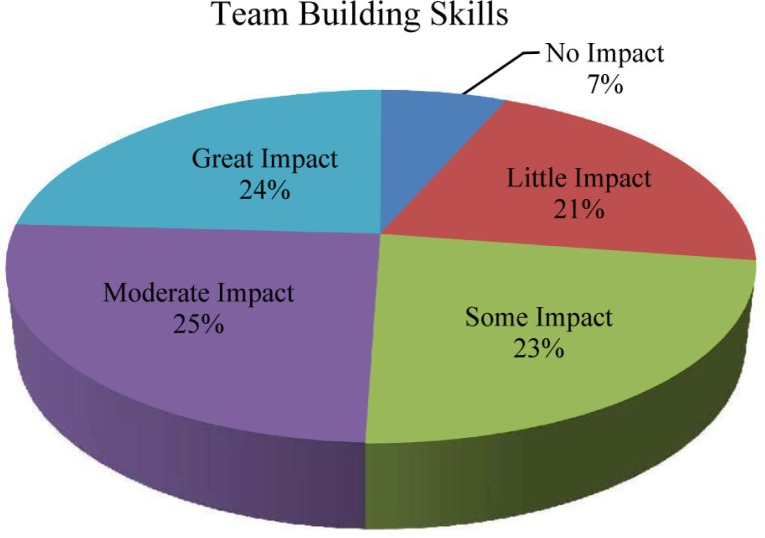
Self-reported impact of the EMT curriculum and ambulance experiences on students’ team-building skills (*N*=97).

We analyzed the Time 3 survey data at the end of the students’ first year. Students were asked to rate the impact of the EMT curriculum and ambulance experiences on their patient care and team-building skills over the past year. Seventy-six students (84%) reported that the EMT curriculum had ‘some impact’ to ‘great impact’ on their patient care skills (Fig. 1), while 66 students (72%) reported that the EMT curriculum had ‘some impact’ to ‘great impact’ on their team-building skills (Fig. 2).

### Qualitative data

The qualitative data that was gathered from student responses to open-ended questions (Time 2 and Time 3 surveys) supported the quantitative data that focused on students’ perceived confidence in early patient interactions, preparation for their Initial Clinical Experience (ICE) and team-building skills (Table 3).

**Table 3:** Qualitative themes and student quotes time 2 (9 weeks) and time 3 (50 weeks)

Early patient interactions	*The EMT curriculum and ambulance experiences have pushed me to approach each patient with an open mind and without bias or preconceptions. It has primed me to consider the fundamentals of medicine, such as circulation and respiration, and to approach patients without jumping to conclusions about diagnoses*. *They've made me comfortable with the basic skills necessary to take care of patients in a clinical setting (e.g., taking vital signs), and also helped me become comfortable with the elements of a patient history. It's good to be exposed to a tense environment* *I have more confidence when talking to patients, after spending time on the ambulances you get used to having Conversations with strangers and it prepares you to act even in situations where you may feel nervous or uncomfortable. I think I am more assertive in ICE than I would have been otherwise. I also felt more comfortable with the basics of a physical exam (pulse, blood pressure, respiratory rate, etc.) than I would have otherwise*.
Preparation for Initial Clinical Experience (ICE)	*I have a greater appreciation for what goes on in the pre-hospital setting and it has prepared me to interact with patients in the clinical setting as well. I had immediate patient contact, and my experience talking to patients in ambulances helped me to become more comfortable talking to patients in ICE*. *I think the ambulance experiences have helped me in ICE because they have gotten me more comfortable with talking to patients. Also, it gave me some experience in presenting my findings, such as BP, pulse, and respiratory rate, which I think helped me initially in my ICE experiences to make a good positive first impression* *The EMT shifts provide an opportunity to display you are not just another first-year medical student which often is a problem at the beginning of ICE*. *Having confidence in interacting with patients and having the title of EMT has translated to confidence that my preceptors have in me. This has resulted in opportunities that may not have occurred otherwise*. *The EMT curriculum helped me to learn how to ask better questions, to not be afraid to admit something I didn't know, and to balance my role as student and learning how to be an emerging physician*.
Team building	*It is important to know now what is going on before the patient arrives in the ER. Because medicine is moving toward a ‘team approach’ to care, it is important to understand how each person works within the system. This way information can be exchanged between the EMT and the physician or ER nurse and not lost in the shuffle. More attention and knowledge of the system leads to better patient care*. *Big picture view of what goes into a patient's medical care, on multiple levels, and how these different areas work together and the need for improvement in communication between these levels*.
Transition from person to professional	*Good transition from bystander to active participant in the medical field*. *The certification enabled us to have some credibility and taught us the basics of interacting with patients in a medical setting*.
Care in diverse settings across a continuum	*I think the EMT curriculum/ambulance experiences did impact my ICE [initial clinical experience] experiences reminding me that the patients we see in our ICE offices go home and have lives beyond those 15–60 minutes that we see them in the office and have to live with their medical conditions all the time*. *It also gives us insight into our patient's life at home, including daily habits and living conditions*. *It can be easy to think of the patient as a case to figure out in the office, but remembering back to when I went into people's dwellings, saw where they live their lives, where they keep their medications, where they have their meals, it helps me to see my role better as one part of their larger life*. *I think that my understanding and appreciation for patients outside of the hospital or office setting was forever changed by my ambulance experiences. Seeing a patient only in the settings that a physician gets to see them in is severely limiting in its context. Like with the rest of medicine, it is almost impossible to diagnose without contextual history, it is also difficult to assess the person as an entirety and a human life without seeing them in their day to day setting*. *The EMT experience gave me an appreciation for this difficulty that I may never have realized without it*.

Additional themes specific to students’ transition from lay person to medical professional were identified, such as the ability to act in emergencies and understanding their roles and expectations as a professional (Table 3).

Time 3 data (50 weeks) continued to support the students’ view that the EMT curriculum was preparatory for their ICE experience in year 1. In addition, a new theme emerged specific to students’ ongoing patient care experiences in diverse settings, which, in addition to the pre-hospital setting reflected the full continuum of care (Table 3).

## Discussion

After completing the CPR course and receiving New York State EMT certification, students continue to have a wide variety of clinical experiences during their first year of medical school. Most notably are their weekly preceptorships in multiple clinical settings including internal medicine, surgery and OB/GYN practices as well as additional scheduled ambulance tours working as an EMT. Students also participate in standardized patient encounters and simulation exercises every 2–3 months throughout the year as a way of continually practicing and demonstrating the ‘knowledge in action’ concepts begun in the CPR course with the EMT curriculum. Based on our longitudinal survey data, students reported the EMT experience as a positive impact on their development and confidence in providing direct patient care skills and were able to relate this confidence to the initial approaches learned during their EMT curriculum. The open-ended comments at Time 3 supported the notion that the skills learned in EMT training are transportable and adaptable to multiple clinical settings and provide an important framework for continued skill building. During the survey development phase, Health Systems residents reported on their prior EMT experiences and held similar thoughts to the students regarding the ability to provide patient care across multiple settings. They stated that these are skills that ‘every doctor should know’ and form a basis for the development of clinical expertise in diverse specialties and diverse clinical settings. An additional benefit expressed by students, specifically in Time 3 data collection, was seeing patients in their home environment prior to hospital care. This was a guiding principle the SOM leadership discussed as one of the goals in the planning of this course.

We were not able to demonstrate that early EMT experiences promoted a significant difference in overall team-building skills. One explanation may be secondary to the fact that our students reported a high level of confidence in this area at baseline and therefore, with the ceiling effect, it would have been very difficult to show a positive change with our sample size. Another possibility is that our students perceived themselves as participants on the EMT team without the necessary experience to be full team members. Of note the two elements of the teambuilding survey that showed statistical improvement were ‘Understanding my role and responsibilities as a medical student’ and ‘Understanding the roles and responsibilities of others’. More advanced skills such as ‘Conflict management’ and ‘Anticipating the needs of other team members’ may not have been reasonable expectations after 9 weeks of medical school and therefore our Team-Building Skills survey may not have asked the right questions for this point in time.

The full impact of providing an EMT curriculum with its associated clinical experience in the first year of medical school cannot be fully understood without acknowledging the expression of the students’ experiences in their own words. The qualitative data provides insight into the students’ development of confidence and professional attitudes as they subsequently cared for patients in the community. In addition to acquiring important skills for their practice as physicians, the EMT curriculum has given our students first-hand experience with a very important part of the health care continuum of which most physicians have little understanding or knowledge.

### Limitations

As the first medical school in the USA to implement an intensive EMT certification course incorporated into a basic science curriculum appropriate for medical student education, we have no comparison data to other medical schools. The small number of students (*n*=13) who were EMTs prior to entering medical school did not allow a subgroup analysis of this population. Furthermore, we had to develop a new survey as there is no prior research on the impact of EMT training within a medical school curriculum. A final limitation was the variation in the experiences the students had during ambulance tours. An EMT shift is unpredictable and the types of cases encountered are not uniform, resulting in variable patient care experiences. Thus far, we have only had the opportunity to assess the impact of this early legitimate clinical experience on our first two classes of students in the first and second year of their medical education.

### Future investigation

We anticipate that the early clinical skills developed during the EMT experiences will be a continued resource to students as they advance in their clinical education during year 3 and 4 as well as in their future residencies. We plan to continue our research and follow this cohort of students longitudinally into their graduate medical education and training to further assess the impact of this innovative curriculum.

## Conclusions

The purpose of this manuscript is to report on the impact of an EMT curriculum as a way to provide early meaningful clinical experience for first year medical students. Providing a legitimate clinical role for students builds levels of confidence in patient care and team-building skills over the first year of medical education. We hypothesized that beyond the development of an early clinical approach to patients, this novel course would 1) increase students’ confidence in their ability to perform patient care skills in multiple care settings and 2) that early exposure to patient care, through involvement with EMS, would enhance students’ comfort with team-building skills early in their training. The integration of an enhanced EMT curriculum into the first course at Hofstra North Shore-LIJ School of Medicine provided students with a significant increase in their confidence to perform patient care skills. More importantly, at the end of their first year of medical school, students reported that confidence gained in their EMT clinical experience persisted in diverse care settings, including community-based practices and during continued EMT ambulance tours. We were not, however, able to demonstrate a significant difference in team-building skills.
